# Supplementary material for: Rare genomic copy number variants implicate new candidate genes for bicuspid aortic valve
Source: PLoS One. 2024 Sep 6;19(9):e0304514. doi: 10.1371/journal.pone.0304514 (PMC11379187; doi:10.1371/journal.pone.0304514)
Supplement: S1 Appendix — (DOCX) [file pone.0304514.s001.docx]

Export microarray data from GenomeStudio

- 1. In Samples Table, set filter to Call Rate > 0.95 !Gender = Unknown
  2. In SNP Table, set filter to GenTrainScore > 0
  3. Create Final Report:
     1. Include intensity only SNPs; exclude zeroed SNPs
     2. Include only samples shown in Sample Table
     3. Select the following fields (tab-delimited)

• SampleID

• SNPname (optional if a separate probe file is provided)

• Chr (optional if a separate probe file is provided)

• Position (optional if a separate probe file is provided)

• allele1-Forward (or allele1-Top)

• allele2-Forward (or allele2-Top)

• X

• Y

• Log R Ratio

• B allele Freq

- 1. This creates a tab-delimited text file {dataset}.final.report.txt with the data for analysis
  2. Export a list of SNP names positions to {dataset}.snp.txt
  3. Export a list of sample genders to {dataset}.gender.txt

cnvPartition (cnvp) analysis (GenomeStudio plugin)

1. CNVP settings: confidence 50, minsnps 6, False, False, True, True
2. Export calls as .txt file

PennCNV (penn) analysis

1. Split GenomeStudio data file into individual call files:

perl split_illumina_report.pl --prefix splitfiles/ {dataset}.final.report.txt

1. Create .pfb file:

perl compile_pfb.pl --snpposfile {dataset}.snp --listfile {dataset}.txt --output {dataset}.pfb

1. Detect CNVs:

perl detect_cnv.pl -test -hmm hhall.hmm --pfbfile {dataset}.pfb –listfile {dataset}.txt --log {dataset}.log --out {dataset}.cnv --gcmodelfile hgXX.gcmodel ---minsnp 6 --confidence

1. Remove CNVs that overlap with polymorphic regions:
   1. scan_region.pl {dataset}.cnv excluderegions --minqueryfrac 0.5 > excluded
   2. fgrep -v -f excluded {dataset}.cnv > {dataset}.scan.cnv
2. Clean up CNV calls:

perl clean_cnv.pl --signalfile --bp combineseg {dataset}.scan.cnv

QuantiSNP (qsnp) analysis

1. Split GenomeStudio data file into individual call files using EnsembleCNV:

perl 01_initial_call/prepare_IPQ_input_file/finalreport_to_QuantiSNP.pl -prefix {dataset}/ -suffix .txt {dataset}.final.report.txt

1. Call individual samples (script file):

#!/bin/bash

OUTPUTDIR="/{dataset}/results"

QUANTIDIR="/quantisnp/2.3"

PROJECTNAME="{dataset}"

USERNAME="{username}”

for FILE in ` find ${OUTPUTDIR} -type f -name "*.txt" `; do

FILENAME=` basename $FILE `

BASENAME=${FILENAME::-4}

# Check the output from these echo commands to make sure script is parsing as expected

echo -n "FILE = "

echo $FILE $BASENAME

echo "sh /quantisnp/2.3/linux64/run_quantisnp2.sh /quantisnp/MCR/v79 --outdir ${OUTPUTDIR} --levels ${QSDIR}/config/levels.dat --config ${QSDIR}/config/params.dat --sampleid ${BASENAME} --input-files ${OUTPUTDIR}/${BASENAME}.txt --logfile ${OUTPUTDIR}/${PROJECTNAME}.log --plot --gcdir [genome_version] --verbose --doXcorrect" >> ${USERNAME}.Quantisnp.script

done

1. Combine individual sample calls (script file):

#!/bin/bash

for FILE in ` find ${OUTPUTDIR}/results -type f -name "*.cnv" `; do

FILENAME=` basename $FILE `

BASENAME=${FILENAME::-4}

# Check the output from these echo commands to make sure script is parsing as expected

echo -n "FILE = "

echo $FILE $BASENAME

tail -n +2 ${OUTPUTDIR}/results/${BASENAME}.cnv >> ${OUTPUTDIR}/combined/{dataset}.quantisnp.cnv

done

Combine individual CNV calls into consensus call list

- - - 1. Convert all outputs to PLINK format:
         1. penn: awk '{print $5,$5,$1,$2,$3,$4,$8,$9}'
         2. qsnp: use maximum Bayes score/awk '{print $1,$1,$2,$3,$4,$9,$10,$8}'
         3. cnvp: no sites: awk '{print $1,$1,$3,$4,$5,$7,$10,"0"}'
      2. Remove X Y chromosome calls: awk '{if ($3<23) print $0}'
      3. Convert all autosomal calls to CN=3 or CN=1:
         1. awk '{if ($6>3) {print $1,$2,$3,$4,$5,3,$7,$8} else {print $1,$2,$3,$4,$5,$6,$7,$8}}’
         2. awk '{if ($6<1) {print $1,$2,$3,$4,$5,1,$7,$8} else {print $1,$2,$3,$4,$5,$6,$7,$8}}'
      4. Create *.map *.fam files using PLINK
      5. Remove large CNVs: > 5 Mb, small CNVs: < 20 Kb or < 6 SNPs, CNVs with score < 20:

$PLINK -noweb -allow-no-sex -cnv-list *.cnv -map *.cnv.map -fam *.fam -cnv-kb 20 -cnv-max-kb 5000 -cnv-sites 6 -cnv-score 20 -cnv-write -out *.qc1

- - - 1. Remove CNVs with score < 100 size > 500 Kb:
         1. awk ‘{print $1,$2,$3,$4,$5,$5-$4,$6,$7,$8}’ *.qc1.cnv > *.qc2.cnv
         2. awk ‘{if (($6>500000) && ($8<100)) print $0 }’ *.qc2.cnv > *.low_score.cnv
         3. grep -vf *.low_score.cnv *.qc2.cnv > *.qc3.cnv
         4. awk ‘{print $1,$2,$3,$4,$5,$7,$8,$9}’ *.qc3.cnv >*.qc4.cnv
      2. Merge CNV calls:
         1. Create pairwise call overlap files:

cat *qc4.cnv > *.all.qc4.cnv

$PLINK --cnv-make-map & --cnv-check-no-overlap: > *.all.qc4.cnv.overlap

- 1. Combine all overlapping CNVs into one file:
     1. tr -s “ “ < *.all.qc4.cnv.overlap > g; mv g *.all.qc4.cnv.overlap
     2. tr “\t” “ “ < *.qc4.cnv > g; mv g *.qc4.cnv
     3. tr -s “ “ < *.qc4.cnv > g; mv g *.qc4.cnv
     4. sed “s/^[ \t]*//” *.all.qc4.cnv.overlap > g; mv g *.all.qc4.cnv.overlap
     5. grep -f *.all.qc4.cnv.overlap *all.qc4.cnv > *.all.qc4.overlap.cnv
  2. Add top-scoring penncnv calls:
     1. wc -l *.penn.qc4.cnv => X
     2. sort -k7nr *.penn.qc4.cnv | head -(0.1X) > *.penn.top10.cnv
     3. cat *.penn.top10.cnv *.all.overlap.cnv > *.final.overlap.cnv

1. Replace SNP coordinates with SNP names:
2. cut -d’ ‘ -f3,4 *.final.overlap.cnv > *.startcoords; cut -d’ ‘ -f3,5 *final.overlap.cnv > *.endcoords
3. Create *.snps file from *.snp.txt files exported from GenomeStudio:
   1. cat [X].snp.txt [Y].snp.txt [Z].snp.txt > *.snps
   2. sort -u -k1 -k2n -k3n *.snps > g; mv g *.snps
4. awk '{print "sed -i \047s/"$2,$3"/"$1"/g\047 *.startcoords"}' *.snps > all.snp.sh
   1. grep -f *.[start/end]coords *.snp.sh > *.[start/end].sh
   2. sed -i ‘s/start/end/g’ *.end.sh
   3. bash *.[start/end].sh
5. Reformat *.final.overlap.cnv file into penncnv format:
   1. awk '{if ($6<3) print $1,$2,$3,$4,$5,"state2,cn=1",$7,$8; else print $1,$2,$3,$4,$5,"state5,cn=3",$7,$8;}' *.final.overlap.cnv > *.penn1
   2. awk '{print "chr",$3,":",$4,"-",$5,"numsnp=",$8,"length=",$5-$4,$6,$1,"startsnp=","endsnp=","conf=",$7}' *.penn1 > *.penn2
   3. cut -d' ' -f1-13 *.penn2 > *.penn3
   4. paste -d' ' *.penn3 *.startcoords > *.penn4
   5. cut -d' ' -f14 *.penn2 > *.penn5
   6. paste -d' ' *.penn4 *.penn5 *.endcoords > *.penn6
   7. cut -d' ' -f15-16 *.penn2 > *.penn7
   8. paste -d' ' *.penn6 *.penn7 > *.final.overlap.penn.cnv
   9. sed -i 's/ - /-/g' *.final.overlap.penn.cnv
   10. sed -i 's/ : /:/g' *.final.overlap.penn.cnv
   11. sed -i 's/= /=/g' *.final.overlap.penn.cnv
   12. sed -i 's/chr /chr/g' *.final.overlap.penn.cnv
6. Merge overlapping CNV fragments:
   1. perl clean_cnv.pl --signalfile *.snps --bp combineseg *.final.overlap.penn.cnv > *.final.cleancnv1
   2. Re-run step ‘a’ until all overlapping fragments are merged (3-4 iterations)
7. Convert to PLINK format:
   1. perl penncnv_to_plink.pl -i *.final.cleancnv1 -o *.final.cnv1
   2. Run PLINK ‘--check-no-overlap’ again
   3. Remove any overlapping calls that remain > *.final.cnv2

Sample-level Quality Control

- - - 1. Calculate mean SD of CNV calls per dataset using penncnv *.indiv file:

1. tr -s “ “ < *.indiv | sort -k4nr >g; mv g *.indiv
2. Calculate mean SD of number of CNVs:
   - 1. Mean: cut -d’ ‘ -f5 *.indiv > *.numcnv; sort -n *.numcnv | awk -f calculate.awk
     2. SD: awk '{delta = $1 - avg; avg += delta / NR; mean2 += delta * ($1 - avg); } END { print sqrt(mean2 / NR); }' *.numcnv
3. Remove samples with high LRRSD or excessive CNV calls:
   1. awk '{if ($4<0.35&&$10<X) print $0}' *.qc.txt > *.qc.pass

where X=2xSD+mean numCNV

- 1. cut -f1 *.qc.pass > g; mv g *.qc.pass
  2. tr “\t” “ “ < *.final.cnvX > g; mv g *.final.cnv2
  3. grep -wf *.qc.pass *.final.cnv2 > *.final.cnv3

1. Review any duplicate samples
2. Separate large CNVs:
   1. awk '{if (($5-$4)>1000000) print $1,$3,$4,$5-$4,$6,$7}' *.CNVP.cnv > *.CNVP.large.cnv
   2. grep -wf *.qc.pass *.CNVP.large.cnv > g; mv g *.CNVP.large.cnv
   3. cut -d' ' -f1-3 *.CNVP.large.cnv > *.large.segs
   4. grep -f *.large.segs *.CNVP.large.cnv | sort -k7nr > *.large.cnv

Curate rare CNVs in PLINK (<https://zzz.bwh.harvard.edu/plink/cnv.shtml>)

1. Large genic CNVs:

--cnv-kb 250 + –cnv-max-kb 5000 + --cnv-intersect

1. Rare genic CNVs (X~1:1000):

--cnv-freq-exclude-above X + --cnv-intersect

1. Genic CNVs unique to cases:

--cnv-unique + –cnv-intersect + --cnv-write, followed by --filter-cases

1. Case-control tests of large rare genic CNVs:

--mperm 10000 + --cnv-intersect + --cnv-test-region

1. Case-control tests of CNV burden:

--cnv-indiv-perm + --cnv-count + --mperm

1. Enrichment for genic CNVs:

--cnv-count + --cnv-enrichment-test

1. Enrichment for pathway genes in genic CNVs:

--cnv-intersect + --cnv-write followed by --cnv-count + --cnv-enrichment-test

1. Reformat PLINK *.reg file as single line per gene:
   1. tr "\n" " " < *.reg > g; mv g *.reg
   2. tr -s " " < *.reg > g; mv g *.reg
   3. sed -i “s/ FID IID PHE CHR BP1 BP2 TYPE KB OLAP OLAP_U OLAP_R //g” *.reg
   4. sed -i "s/RANGE (+\/- 20kb ) //g" *.reg
   5. tr "[" "\n" < *.reg > g; mv g *.reg
