# Supplementary material for: Rare genomic copy number variants implicate new candidate genes for bicuspid aortic valve
Source: PLoS One. 2024 Sep 6;19(9):e0304514. doi: 10.1371/journal.pone.0304514 (PMC11379187; doi:10.1371/journal.pone.0304514)
Supplement: S1 Table — WLS, Wisconsin Longitudinal Study on Aging; HRS, Health and Retirement Study; Accession, accession number in the Database of Genotypes Phenotypes. WLS includes data on a cohort of 10,300 individuals who graduated from Wisconsin high schools in 1957. HRS includes data on 37,000 individuals aged 50 above from 23,000 households across the United States. (DOCX) [file pone.0304514.s002.docx]

| Cohort | Study | Samples | Accession | Microarray |
| --- | --- | --- | --- | --- |
| WLS | Wisconsin Longitudinal Study on Aging | 8969 | phs001157.v1.pl | Illumina HumanOmniExpress-24 v1.1 |
| HRS | Health Retirement Study | 9426 | phs000428.v2.pl | Illumina Human Omni2.5-Quad |
