# Supplementary material for: Rare genomic copy number variants implicate new candidate genes for bicuspid aortic valve
Source: PLoS One. 2024 Sep 6;19(9):e0304514. doi: 10.1371/journal.pone.0304514 (PMC11379187; doi:10.1371/journal.pone.0304514)
Supplement: S2 Table — Used for intersection studies. (DOCX) [file pone.0304514.s003.docx]

| **BAV** | **CHD** | **CHD** | **CHD** | **CHD** | **CHD** | **CHD** | **CHD** |
| --- | --- | --- | --- | --- | --- | --- | --- |
| CELSR1 | ABCC9 | CFC1 | FOXC1 | KAT6B | NIPBL | RANBP9 | TBX1 |
| GATA4 | ACTB | CFL1 | FOXC2 | KDM6A | NOTCH2 | RBM10 | TBX2 |
| GATA5 | ACVR1 | CHD4 | FOXF1 | KDR | NPHP3 | RBPJ | TBX3 |
| GATA6 | ACVR2B | CHD7 | FOXH1 | KIF7 | NR1D2 | RECQL4 | TBX5 |
| MAT2A | ADAM17 | CITED2 | FOXJ1 | KMT2D | NR2F2 | RFX3 | TFAP2B |
| MATR3 | ADAMTS10 | CREBBP | FOXP1 | KRAS | NRAS | RHOA | TP53 |
| MIB1 | AKAP13 | CRELD1 | FUZ | LATS2 | NSD1 | RHOV | UBC |
| NKX2-5 | AKT1 | CSK | G6PC3 | LRP2 | OFD1 | RIT1 | UBR1 |
| NOTCH1 | APC | CTNNB1 | GALNT1 | LTBP1 | PABPC1 | ROCK1 | UBR4 |
| ROBO4 | ARHGAP31 | DCHS1 | GDF1 | MAML1 | PBX1 | ROCK2 | VEGFA |
| SMAD6 | ARHGEF11 | DDX10 | GJC1 | MAP2K1 | PCDHGA2 | ROR2 | YWHAB |
| TBX20 | ARID1B | DDX3X | GLRX3 | MAP2K2 | PCSK5 | RPL5 | ZBTB14 |
|  | B3GALTL | DHCR7 | GRB2 | MAP3K5 | PCSK6 | RPS19 | ZEB2 |
|  | B3GAT3 | DLL4 | HAND1 | MAPK1 | PDK1L1 | RPS27A | ZFPM1 |
|  | BBS2 | DNAH5 | HAND2 | MEGF8 | PKD1 | SALL1 | ZFPM2 |
|  | BBS6 | DOCK6 | HDAC10 | MEIS2 | PRDM1 | SHOC2 | ZIC3 |
|  | BCL9 | E2F6 | HES1 | MGRN1 | PRICKLE1 | SLIT3 |  |
|  | BCOR | EFTUD2 | HEY2 | MINK1 | PRKACB | SMARCA4 |  |
|  | BMP1 | EHMT1 | HIC2 | MKKS | PRKCD | SMARCC1 |  |
|  | BMP4 | ELN | HOXA1 | MNDA | PRKCH | SMURF1 |  |
|  | BMPR1A | EOGT | HRAS | MSX1 | PTK7 | SON |  |
|  | BMPR2 | EP300 | HSP90AB1 | MYH6 | PTPN11 | SOS1 |  |
|  | BRAF | EPHA2 | IGF1R | NAA15 | PYGL | SRC |  |
|  | CAV1 | ESCO2 | ITPR3 | NCK1 | PYGO | SSH2 |  |
|  | CBP | ETS1 | JAG1 | NEK8 | RAB23 | STRA6 |  |
|  | CCDC91 | EVC | JARID2 | NF1 | RAC1 | SUFU |  |
|  | CDH2 | EVC2 | JMJD6 | NFATC1 | RACK1 | TAB1 |  |
|  | CDK13 | FGF19 | KANSL1 | NGFR | RAF1 | TAB2 |  |
|  | CDK4 | FLT4 | KAT6A | NIFK | RAI1 | TBC1D32 |  |
