# Supplementary material for: Rare genomic copy number variants implicate new candidate genes for bicuspid aortic valve
Source: PLoS One. 2024 Sep 6;19(9):e0304514. doi: 10.1371/journal.pone.0304514 (PMC11379187; doi:10.1371/journal.pone.0304514)
Supplement: S3 Table — EBAV, early onset bicuspid aortic valve cohort; BAVGWAS, Genome-wide Association Study from the International BAV Consortium; WLS, Wisconsin Longitudinal Study on Aging; HRS, Health Retirement Study; PennCNV, number of CNV calls detected by PennCNV algorithm after quality control; cnvPartition, number of CNV calls detected by cnvPartition algorithm after quality control; QuantiSNP, number of CNVs detected by QuantiSNP algorithm after quality control; Merged, number of CNV regions after merging adjacent calls; >5 MB, number of CNV regions that are larger than 5 megabases; Rare, number of CNVs that occur in less than 1 in 1000 samples of the combined datasets; Rare Deletions, number of large (> 250 Kb) rare deletions. (DOCX) [file pone.0304514.s004.docx]

|  | EBAV | BAVGWAS | WLS | HRS |
| --- | --- | --- | --- | --- |
| PennCNV | 6781 | 73784 | 58115 | 163938 |
| cnvPartition | 2289 | 33640 | 31148 | 51794 |
| QuantiSNP | 1798 | 21326 | 14346 | 85312 |
| Merged | 902 | 7622 | 21343 | 14657 |
| Deletions | 610 | 2772 | 8170 | 6770 |
| >5 Mb | 9 | 22 | 9830 | 6114 |
| Rare | 84 | 579 | 1443 | 1372 |
| Rare Deletions | 59 | 181 | 285 | 394 |
