# Supplementary material for: Rare genomic copy number variants implicate new candidate genes for bicuspid aortic valve
Source: PLoS One. 2024 Sep 6;19(9):e0304514. doi: 10.1371/journal.pone.0304514 (PMC11379187; doi:10.1371/journal.pone.0304514)
Supplement: S4 Table — Gene(s), genes intersected by CNV; Chr, chromosome; Start BP, start base pair of CNV; Stop BP, stop base pair of CNV; DUP, duplication; DEL, deletion. All CNVs were validated by direct inspection in GenomeStudio. (DOCX) [file pone.0304514.s005.docx]

| Gene(s) | Chr | Start | Stop | Type |
| --- | --- | --- | --- | --- |
| None | 1 | 187296703 | 187609850 | DEL |
| *HYDIN, NBPF1, LOC78989, NBPF13P, PRKAB, PDIA3P, FM05, CHD1L, LINC0064, BCL9, ACP6, GJA5* | 1 | 146326373 | 147340734 | DUP |
| *ELTD1* | 1 | 79238015 | 79619893 | DEL |
| *HIST3HA, HIST3HBB, MIR4666A, BC015435, RNF187, DUSP5P1,* *RHOU* | 1 | 228625778 | 228880626 | DUP |
| *SLC35F5, MIR478, ACTR3, LOC100499194,* *LOC440900* | 2 | 114458921 | 115208197 | DUP |
| *LOC7798* | 2 | 4638261 | 5564549 | DUP |
| *ZNF860,* *GPD1L* | 3 | 31901848 | 32165994 | DUP |
| *KCNH8* | 3 | 19363589 | 19813225 | DEL |
| *BC005018*, *AK09585* | 4 | 84658825 | 85270309 | DUP |
| None | 5 | 25468811 | 25719474 | DEL |
| *ARSB* | 5 | 78016365 | 78286867 | DUP |
| *MANEA* | 6 | 95836160 | 96095769 | DEL |
| None | 8 | 89353386 | 89800669 | DEL |
| *BC045738, AK18880* | 8 | 2319555 | 2585105 | DUP |
| None | 8 | 4201652 | 4493979 | DUP |
| *MRSA, AK30707, PRSS55, RP1L1, C8orf74, SOX7, BC043573, PINX1, MIR13, AK09455, MTMR9, CR749668, SLC35G5, TDH, BC038546, C8orf1, FAM167A, BLK, LINC0008, GATA4, C8orf49, NEIL, FDFT1, CTSB, DEFB136, DEFB135, DEFB134* | 8 | 10111571 | 11856864 | DUP |
| *TINKS, MIR597* | 8 | 9368431 | 9745798 | DUP |
| *GATA4, C8orf49, NEIL, FDFT1, CTSB* | 8 | 11448529 | 11732454 | DUP |
| *GATA4, C8orf49, NEIL, FDFT1, CTSB* | 8 | 11448529 | 11808756 | DUP |
| *INPP5A, NKX6-, TTC40, LOC39989, GPR13, CS330190, KNDC1, UTF1, VENTX, MIR0, ADAM8, TUBGCP, ZNF511, PRAP1, BC04794, CALY, FUOM, ECHS1, MIR3944,* *PAOX* | 10 | 134505252 | 135203544 | DEL |
| None | 12 | 84108147 | 84443245 | DUP |
| *KLHL1, ATXN8OS, LINC00348* | 13 | 70578273 | 71593281 | DUP |
| *ARHGAP11A, SCG5, C15orf45, AX747968, GREM1, FMN1, TMCO5B, RYR3, AVEN, M5, EMC7, PGBD4, KATNBL1, EMC4, SLC1A6, NOP10, NUTM1, LPCAT4, GOLGA8A, MIR133, KIAA0855* | 15 | 32908301 | 34761123 | DEL |
| *MIR318, HSBP1, MLYCD, OSGIN1, NECAB* | 16 | 83302526 | 84016062 | DUP |
| *DOCB, FLJ44838, RPH3AL, MGC70870, LOC100506388, C17orf97, FAM101B,* *VPS53* | 17 | 1389 | 582832 | DEL |
| None | 18 | 57590566 | 57955945 | DUP |
| *PCP4, DSCAM, MIR4760, DSCAM-AS1, PCP4, DSCAM, MIR4760, DSCAM-AS1, PCP4, DSCAM, MIR4760, DSCAM-AS1, PCP4, DSCAM, MIR4760,DSCAM-AS1* | 21 | 41268738 | 41813285 | DUP |
| *PCP4, DSCAM, MIR4760, DSCAM-AS1, PCP4, DSCAM, MIR4760, DSCAM-AS1, PCP4, DSCAM, MIR4760, DSCAM-AS1, PCP4, DSCAM, MIR4760, DSCAM-AS1* | 21 | 41268738 | 41823356 | DUP |
| *PCP4, DSCAM, MIR4760, DSCAM-AS1, PCP4, DSCAM, MIR4760, DSCAM-AS1, PCP4, DSCAM, MIR4760, DSCAM-AS1, PCP4, DSCAM, MIR4760,* *DSCAM-AS1* | 21 | 41278694 | 41823356 | DUP |
| *PCP4, DSCAM, MIR4760, DSCAM-AS1, PCP4, DSCAM, MIR4760, DSCAM-AS1, PCP4, DSCAM, MIR4760, DSCAM-AS1, PCP4, DSCAM, MIR4760, DSCAM-AS1* | 21 | 41278694 | 41823356 | DUP |
| *SEPT5-GP1BB, TBX1, GNB1L, Corf9, TXNRD, COMT, MIR4761, ARVCF, TANGO, MIR185, DGCR8, MIR3618, MIR1306, TRMTA, RANBP1, ZDHHC8, LOC388849, LOC84865, LINC00896* | 22 | 19580050 | 20227551 | DUP |
| *WNT7B, LOC730668, LINC00899, Corf6, LOC150381, MIRLET7BHG, LOC554174, MIR3619, MIRLET7A3, MIR4763, MIRLET7B, PPARA, CDPF1, PKDREJ, TTC38, GTSE1-AS1, GTSE1, BC0691, BC0134, TRMU, CELSR1, GRAMD4, CERK, TBC1D22A, FLJ32756, BC037972, LINC00898, BC039485, AK093107, MIR3201, FAM19A5, LOC84933, MIR4535, LOC10018946, BC033837, Corf34, BRD1, LOC90834, ZBED4, ALG1, CRELD, PIM3, IL17REL, MLC1, MOV10L1, PANX, TRABD, SELO, TUBGCP6, HDAC10, MAPK1, MAPK11, PLXNB, DENND6B, PPP6R, AB3777, SBF1, ADM, MIOX, LMF, NCAPH, SCO, TYMP, ODF3B, KLHDC7B, SYCE3, CPT1B, CHKB-CPT1B, BC04819, CHKB, CHKB-AS1, MAPK8IP, ARSA, SHANK3, BC050343,* *ACR* | 22 | 46261909 | 51187440 | DEL |
