# Supplementary material for: Rare genomic copy number variants implicate new candidate genes for bicuspid aortic valve
Source: PLoS One. 2024 Sep 6;19(9):e0304514. doi: 10.1371/journal.pone.0304514 (PMC11379187; doi:10.1371/journal.pone.0304514)
Supplement: S5 Table — CNV, samples with large rare CNVs; n, number of samples; TAA, thoracic aortic aneurysm; AR, aortic regurgitation; AS, aortic stenosis; Other lesions, other congenital heart malformations; >1 Affected, number of families with more than one affected individual. Percentages are in parentheses. *Significantly increased. (DOCX) [file pone.0304514.s006.docx]

|  | CNV (n=28) | No CNV (n=244) |
| --- | --- | --- |
| Female | 11 (39) | 85 (35) |
| Average Age of Onset (years) | 10±10* | 37±21 |
| TAA | 1 (4) | 37 (15) |
| Predominant AR | 4 (14) | 35 (14) |
| Predominant AS | 3 (13) | 47 (19) |
| Other Lesions | 8 (29) | 51 (21) |
| Aortic Valve Surgery | 9 (32) | 82 (34) |
| Families with >1 Affected | 4 (14)* | 11 (4.5) |
