# Supplementary material for: Rare genomic copy number variants implicate new candidate genes for bicuspid aortic valve
Source: PLoS One. 2024 Sep 6;19(9):e0304514. doi: 10.1371/journal.pone.0304514 (PMC11379187; doi:10.1371/journal.pone.0304514)
Supplement: S6 Table — Chr, Chromosome; EMP1, permutation-based empiric P-value; EMP2, after genome-wide correction. *Top candidate genes. (DOCX) [file pone.0304514.s007.docx]

| Gene | Chr | EMP1 | EMP2 |
| --- | --- | --- | --- |
| *PCP4* | 21 | 1.00X10^-4^ | 1.00X10^-4^ |
| *DSCAM* | 21 | 1.00X10^-4^ | 1.00X10-4 |
| *MIR4760* | 21 | 1.00X10^-4^ | 1.00X10-4 |
| *DSCAM** | 21 | 1.00X10^-4^ | 1.00X10-4 |
| *ACTR3* | 2 | 1.00X10^-4^ | 0.0012 |
| *LOC100499194* | 2 | 1.00X10^-4^ | 0.0012 |
| *LOC440900* | 2 | 1.00X10^-4^ | 0.0012 |
| *LOC727982* | 2 | 0.0002 | 0.026897 |
| *SLC35F5* | 2 | 0.0004 | 0.026897 |
| *MIR4782* | 2 | 0.0004 | 0.026897 |
| *DPP10* | 2 | 0.0008 | 0.080092 |
| *GATA4** | 8 | 1.00X10^-4^ | 0.0012 |
| *C8orf49* | 8 | 1.00X10^-4^ | 0.0012 |
| *NEIL2* | 8 | 1.00X10^-4^ | 0.0012 |
| *FDFT1* | 8 | 0.0024 | 0.907809 |
| *CTSB* | 8 | 0.0024 | 0.907809 |
| *LINC00208* | 8 | 0.0002 | 0.026897 |
| *MIR548N* | 2 | 0.0029 | 0.691731 |
| *TTN-AS1* | 2 | 0.0029 | 0.691731 |
| *TTN** | 2 | 0.0029 | 0.691731 |
| *AX746670* | 2 | 0.0029 | 0.691731 |
| *KLHL1* | 13 | 0.004 | 0.691731 |
| *ATXN8OS* | 13 | 0.004 | 0.691731 |
| *COMT* | 22 | 0.0035 | 0.691731 |
| *MIR4761* | 22 | 0.0035 | 0.691731 |
| *ARVCF* | 22 | 0.0035 | 0.691731 |
| *TANGO2* | 22 | 0.0035 | 0.691731 |
| *MIR185* | 22 | 0.0035 | 0.691731 |
| *DGCR8* | 22 | 0.0035 | 0.691731 |
| *MIR3618* | 22 | 0.0035 | 0.691731 |
| *MIR1306* | 22 | 0.0035 | 0.691731 |
| *TRMT2A* | 22 | 0.0035 | 0.691731 |
| *RANBP1* | 22 | 0.0035 | 0.691731 |
| *ZDHHC8* | 22 | 0.0035 | 0.691731 |
| *LOC388849* | 22 | 0.0035 | 0.691731 |
| *HYDIN2* | 1 | 0.006899 | 0.906509 |
| *NBPF12* | 1 | 0.006899 | 0.906509 |
| *LOC728989* | 1 | 0.006899 | 0.906509 |
| *NBPF13P* | 1 | 0.006899 | 0.906509 |
| *PRKAB2* | 1 | 0.006899 | 0.906509 |
| *PDIA3P* | 1 | 0.006899 | 0.906509 |
| *FMO5* | 1 | 0.006899 | 0.906509 |
| *CHD1L* | 1 | 0.006899 | 0.906509 |
| *LINC00624* | 1 | 0.006899 | 0.906509 |
| *BCL9* | 1 | 0.006899 | 0.906509 |
| *ACP6* | 1 | 0.006899 | 0.906509 |
| *GJA5** | 1 | 0.006899 | 0.906509 |
| *PARD3* | 10 | 0.008999 | 0.906509 |
| *NECAB2* | 16 | 0.008299 | 0.906509 |
| *TBX1** | 22 | 0.009699 | 0.906509 |
| *GNB1L* | 22 | 0.009699 | 0.906509 |
| *C22orf29* | 22 | 0.009699 | 0.906509 |
| *TXNRD2* | 22 | 0.009699 | 0.906509 |
| *LOC284865* | 22 | 0.008399 | 0.906509 |
| *LINC00896* | 22 | 0.008399 | 0.906509 |
| *RTN4R* | 22 | 0.008399 | 0.906509 |
| *MIR1286* | 22 | 0.008399 | 0.906509 |
