# Supplementary material for: Rare genomic copy number variants implicate new candidate genes for bicuspid aortic valve
Source: PLoS One. 2024 Sep 6;19(9):e0304514. doi: 10.1371/journal.pone.0304514 (PMC11379187; doi:10.1371/journal.pone.0304514)
Supplement: S7 Table — Gene(s), genes intersected by CNV; Chr, chromosome; Start, start base pair of CNV; Stop, stop base pair of CNV; DUP, duplication; DEL, deletion. *Call in apparently unaffected family member. **Call in affected family member belonging to multiplex family. (DOCX) [file pone.0304514.s008.docx]

| Gene(s) | Chr. | Start BP | Stop BP | Type |
| --- | --- | --- | --- | --- |
| *HYDIN2, NBPF12, LOC728989, NBPF13P, PRKAB2, PDIA3P, FM05, CHD1L, LINC00624, BCL9, ACP6, GJA5* | 1 | 146326373 | 147340734 | DUP |
| *HYDIN2, NBPF12, LOC728989, NBPF13P, PRKAB2, PDIA3P, FM05, CHD1L, LINC00624, BCL9, ACP6, GJA5** | 1 | 146326373 | 147229299 | DUP |
| *MIR4782, SLC35F5, ACTR3, LOC100499194, LOC440900** | 2 | 114426115 | 115208197 | DUP |
| *MIR4782, SLC35F5, ACTR3, LOC100499194, LOC440900** | 2 | 114614021 | 114732241 | DUP |
| *MIR4782, SLC35F5, ACTR3, LOC100499194, LOC440900** | 2 | 114458921 | 115208197 | DUP |
| *MIR4782, SLC35F5, ACTR3, LOC100499194, LOC440900* | 2 | 114458921 | 115208197 | DUP |
| *TTN, AX746670, TTN-AS1, MIR548N* | 2 | 179364778 | 179486671 | DUP |
| *TTN, AX746670, TTN-AS1, MIR548N** | 2 | 179395466 | 179517632 | DUP |
| *MICA* | 6 | 31360255 | 31453029 | DEL |
| *MICA* | 6 | 31360255 | 31485928 | DEL |
| *MICA* | 6 | 31360255 | 31487876 | DEL |
| *MICA* | 6 | 31360255 | 31457633 | DUP |
| *MICA* | 6 | 31361397 | 31453029 | DUP |
| *MICA** | 6 | 31360255 | 31453029 | DEL |
| *MICA** | 6 | 31360255 | 31453029 | DEL |
| *MICA** | 6 | 31360255 | 31453029 | DEL |
| *MICA** | 6 | 31360255 | 31485928 | DEL |
| *MICA** | 6 | 31360255 | 31485928 | DEL |
| *MICA** | 6 | 31360255 | 31485928 | DEL |
| *MICA** | 6 | 31383960 | 31485928 | DEL |
| *MICA** | 6 | 31355260 | 31453029 | DEL |
| *GATA4, C8orf49, NEIL2, FDFT1, CTSB* | 8 | 11506208 | 11786255 | DUP |
| *GATA4, C8orf49, NEIL2, FDFT1, CTSB* | 8 | 11103895 | 11856864 | DUP |
| *GATA4, C8orf49, NEIL2, FDFT1, CTSB* | 8 | 11448529 | 11808756 | DUP |
| *GATA4, C8orf49, NEIL2, FDFT1, CTSB* | 8 | 11448529 | 11732454 | DUP |
| *PARD3* | 10 | 35107733 | 35284461 | DUP |
| *PARD3* | 10 | 35107733 | 35271898 | DUP |
| *KLHL1*, *ATXN8OS* | 13 | 70578273 | 71593281 | DUP |
| *KLHL1*, *ATXN8OS** | 13 | 70589082 | 71548725 | DUP |
| *KLHL1*, *ATXN8OS** | 13 | 70730307 | 70773605 | DEL |
| *NECAB2* | 16 | 83302526 | 84016062 | DUP |
| *NECAB2** | 16 | 83303915 | 83999565 | DUP |
| *PCP4, DSCAM, MIR4760,* *DSCAM-AS1* | 21 | 41278694 | 41823356 | DUP |
| *PCP4, DSCAM, MIR4760,* *DSCAM-AS1* | 21 | 41268738 | 41813285 | DUP |
| *PCP4, DSCAM, MIR4760, DSCAM-AS1* | 21 | 41278694 | 41823356 | DUP |
| *PCP4, DSCAM, MIR4760, DSCAM-AS1*** | 21 | 41278694 | 41813285 | DUP |
| *PCP4, DSCAM, MIR4760, DSCAM-AS1* | 21 | 41268738 | 41823356 | DUP |
| *TBX1, GNB1L, C22orf29, TXNRD2, COMT, MIR4761, ARVCF, TANGO2, MIR185, DGCR8, MIR3618, MIR1306, TRMT2A, RANBP1, ZDHHC8, LOC388849, LOC284865, LINC00896* | 22 | 19580050 | 20227551 | DUP |
| *TBX1, GNB1L, C22orf29, TXNRD2, COMT, MIR4761, ARVCF, TANGO2, MIR185, DGCR8, MIR3618, MIR1306, TRMT2A, RANBP1, ZDHHC8, LOC388849, LOC284865, LINC00896, RTN4R, MIR1286* | 22 | 18877787 | 21461607 | DUP |
