# Supplementary material for: Rare genomic copy number variants implicate new candidate genes for bicuspid aortic valve
Source: PLoS One. 2024 Sep 6;19(9):e0304514. doi: 10.1371/journal.pone.0304514 (PMC11379187; doi:10.1371/journal.pone.0304514)
Supplement: S8 Table — Chr, Chromosome; EMP1, permutation-based empiric P-value; EMP2, after genome-wide correction. *Top candidate genes. (DOCX) [file pone.0304514.s009.docx]

| Gene | Chr. | EMP1 | EMP2 |
| --- | --- | --- | --- |
| *MIR1245A* | 2 | 1.00x10^-4^ | 1.00x10^-4^ |
| *MIR1245B* | 2 | 1.00x10^-4^ | 1.00x10^-4^ |
| *MIR3606* | 2 | 1.00x10^-4^ | 1.00x10^-4^ |
| *TMPRSS11E* | 4 | 1.00x10^-4^ | 1.00x10^-4^ |
| *UGT2B17* | 4 | 1.00x10^-4^ | 1.00x10^-4^ |
| *UGT2B15* | 4 | 1.00x10^-4^ | 1.00x10^-4^ |
| *NPAT* | 11 | 1.00x10^-4^ | 1.00x10^-4^ |
| *ATM* | 11 | 1.00x10^-4^ | 1.00x10^-4^ |
| *C11orf65* | 11 | 1.00x10^-4^ | 1.00x10^-4^ |
| *NANOGNB* | 12 | 0.0003 | 0.012 |
| *NANOG** | 12 | 1.00x10^-4^ | 1.00x10^-4^ |
| *OR11H12* | 14 | 1.00x10^-4^ | 1.00x10^-4^ |
| *LOC642426* | 14 | 1.00x10^-4^ | 1.00x10^-4^ |
| *POTEG* | 14 | 1.00X10^-4^ | 1.00x10^-4^ |
| *P712P* | 14 | 1.00X10^-4^ | 1.00x10^-4^ |
| *DQ595091* | 14 | 1.00X10^-4^ | 1.00X10^-4^ |
| *DQ599717* | 14 | 1.00X10^-4^ | 1.00X10^-4^ |
| *P775P* | 14 | 1.00X10^-4^ | 1.00X10^-4^ |
| *BX248778* | 14 | 1.00X10^-4^ | 1.00X10^-4^ |
| *AK022914* | 14 | 1.00X10^-4^ | 1.00X10^-4^ |
| *BC016035* | 14 | 1.00X10^-4^ | 1.00X10^-4^ |
| *DQ786293* | 14 | 1.00X10^-4^ | 1.00X10^-4^ |
| *LOC101101776* | 14 | 1.00X10^-4^ | 1.00X10^-4^ |
| *BC041856* | 14 | 1.00X10^-4^ | 1.00X10^-4^ |
| *LINC00516* | 14 | 1.00X10^-4^ | 1.00X10^-4^ |
| *BC017398* | 14 | 1.00X10^-4^ | 1.00X10^-4^ |
| *AK056135* | 14 | 1.00X10^-4^ | 1.00X10^-4^ |
| *DQ582484* | 14 | 1.00X10^-4^ | 1.00X10^-4^ |
| *POTEM* | 14 | 1.00X10^-4^ | 1.00X10^-4^ |
| *OR11H2* | 14 | 1.00X10^-4^ | 1.00X10^-4^ |
| *OR4Q3* | 14 | 1.00X10^-4^ | 1.00X10^-4^ |
| *OR4M1* | 14 | 1.00X10^-4^ | 1.00X10^-4^ |
| *OR4N2* | 14 | 1.00X10^-4^ | 1.00X10^-4^ |
| *OR4K2* | 14 | 1.00X10^-4^ | 1.00X10^-4^ |
| *OR4K5* | 14 | 1.00X10^-4^ | 1.00X10^-4^ |
| *OR4K1* | 14 | 1.00X10^-4^ | 1.00X10^-4^ |
| *MAPK1* | 22 | 0.00489951 | 0.465853 |
| *NIPBL** | 5 | 0.0121988 | 0.921008 |
| *AHRR* | 5 | 0.0121988 | 0.921008 |
| *C5orf55* | 5 | 0.0121988 | 0.921008 |
| *EXOC3* | 5 | 0.0121988 | 0.921008 |
| *FLJ00157* | 5 | 0.0121988 | 0.921008 |
| *AK023178* | 5 | 0.0121988 | 0.921008 |
| *PP7080* | 5 | 0.0121988 | 0.921008 |
| *BC013821* | 5 | 0.0121988 | 0.921008 |
|  |  |  |  |
