# Supplementary material for: Rare genomic copy number variants implicate new candidate genes for bicuspid aortic valve
Source: PLoS One. 2024 Sep 6;19(9):e0304514. doi: 10.1371/journal.pone.0304514 (PMC11379187; doi:10.1371/journal.pone.0304514)
Supplement: S9 Table — Gene(s), genes intersected by CNV; Chr, chromosome; Start, start base pair of CNV; Stop, stop base pair of CNV; DUP, duplication; DEL, deletion. (DOCX) [file pone.0304514.s010.docx]

| Gene(s) | Chr | Start | Stop | Type |
| --- | --- | --- | --- | --- |
| *LOC100507334* | 2 | 110852875 | 111406073 | DUP |
| *LOC100507334* | 2 | 110982530 | 112007875 | DUP |
| *MIR128-2* | 3 | 35775249 | 35938795 | DUP |
| *MIR128-2* | 3 | 35775249 | 35938795 | DUP |
| *MIR128-2* | 3 | 35785608 | 35936616 | DUP |
| *TMPRSS11E, UGT2B17, UGT2B15, UGT2B10* | 4 | 69599357 | 69712995 | DUP |
| *AHRR, C5orf55, EXOC3, FLJ00157, AK023178, PP7080, BC013821, LOC100996325, CEP72* | 5 | 323965 | 889536 | DUP |
| *AHRR, C5orf55, EXOC3, FLJ00157, AK023178, PP7080, BC013821* | 5 | 287907 | 602256 | DUP |
| *AHRR, C5orf55, EXOC3, FLJ00157, AK023178, PP7080, BC013821* | 5 | 310925 | 548342 | DUP |
| *AHRR, C5orf55, EXOC3, FLJ00157, AK023178, PP7080, BC013821, LOC100996325, CEP72* | 5 | 426109 | 673408 | DUP |
| *AHRR, C5orf55, EXOC3, FLJ00157, AK023178, PP7080, BC013821, LOC100996325, CEP72* | 5 | 589727 | 701920 | DUP |
| *AHRR, C5orf55, EXOC3, FLJ00157, AK023178, PP7080, BC013821, LOC100996325, CEP72* | 5 | 589727 | 701920 | DUP |
| *NIPBL* | 5 | 36764235 | 37046626 | DUP |
| *NIPBL* | 5 | 36805679 | 37046626 | DUP |
| *NIPBL* | 5 | 36898424 | 37046626 | DUP |
| *NIPBL* | 5 | 36911625 | 37052624 | DUP |
| *SGK223, CLDN23, MFHAS1* | 8 | 8064756 | 11143272 | DUP |
| *SGK223, CLDN23, MFHAS1* | 8 | 8064756 | 11882065 | DUP |
| *SGK223, CLDN23, MFHAS1* | 8 | 8064756 | 8655355 | DUP |
| *SGK223, CLDN23, MFHAS1* | 8 | 8114228 | 8627839 | DUP |
| *SGK223, CLDN23, MFHAS1* | 8 | 8202294 | 8674049 | DUP |
| *SGK223, CLDN23, MFHAS1* | 8 | 8221088 | 8650456 | DUP |
| *CUL5* | 11 | 107755731 | 107965390 | DUP |
| *NANOG NANOGNB* | 12 | 7893437 | 8101326 | DUP |
| *NANOG NANOGNB* | 12 | 7918339 | 8109412 | DUP |
| *NANOG* *NANOGNB* | 12 | 7942473 | 8109412 | DUP |
| *NANOG NANOGNB* | 12 | 7942945 | 8123777 | DUP |
| *NANOG NANOGNB* | 12 | 7942945 | 8105015 | DUP |
| *NANOG NANOGNB* | 12 | 7945559 | 8101326 | DUP |
| *NANOG NANOGNB* | 12 | 7945559 | 8105015 | DUP |
| *NANOG NANOGNB* | 12 | 7945559 | 8105015 | DUP |
| *NANOG NANOGNB* | 12 | 7945559 | 8109412 | DUP |
| *NANOG* *NANOGNB* | 12 | 7945559 | 8130958 | DUP |
| *UBE2MP1, LOC283914, LOC146481, LOC100130700* | 16 | 34355747 | 34740580 | DUP |
| *LOC283914* *LOC146481* | 16 | 34428972 | 34723621 | DUP |
| *LOC283914* | 16 | 34433468 | 34663346 | DUP |
| *FAM101B, VPS53, FAM57A* | 17 | 1389 | 641023 | DUP |
| *FAM101B, VPS53, FAM57A, GEMIN4, DQ581337, DBIL5P* | 17 | 225778 | 906268 | DEL |
| *FAM101B, VPS53, FAM57A, GEMIN4* | 17 | 225778 | 649766 | DUP |
| *FAM101B, VPS53, FAM57A, GEMIN4* | 17 | 238906 | 650372 | DUP |
| *FAM101B, VPS53, FAM57A, GEMIN4, DQ581337, DBIL5P* | 17 | 284614 | 831667 | DUP |
| *RYR1, MAP4K1, EIF3K* | 19 | 38683266 | 39116961 | DUP |
| *RYR1, MAP4K1, EIF3K* | 19 | 38976659 | 39116961 | DUP |
| *RYR1, MAP4K1, EIF3K* | 19 | 38993142 | 39116961 | DUP |
| *RYR1, MAP4K1, EIF3K* | 19 | 38993142 | 39116961 | DUP |
| *CYP2A7, CYP2G1P, CYP2B7P1, CYP2B6* | 19 | 41349732 | 41508557 | DUP |
| *CYP2A7, CYP2G1P, CYP2B7P1, CYP2B6* | 19 | 41350509 | 41600054 | DUP |
| *CYP2A7, CYP2G1P, CYP2B7P1, CYP2B6* | 19 | 41354458 | 41588347 | DUP |
| *CYP2A7, CYP2G1P, CYP2B7P1, CYP2B6* | 19 | 41386035 | 41522338 | DUP |
| *CYP2A7, CYP2G1P, CYP2B7P1, CYP2B6* | 19 | 41386814 | 41531705 | DUP |
| *CYP2A7, CYP2G1P, CYP2B7P1, CYP2B6* | 19 | 41386814 | 41519306 | DUP |
