# Supplementary material for: Rare genomic copy number variants implicate new candidate genes for bicuspid aortic valve
Source: PLoS One. 2024 Sep 6;19(9):e0304514. doi: 10.1371/journal.pone.0304514 (PMC11379187; doi:10.1371/journal.pone.0304514)
Supplement: S10 Table — Chr, chromosome; Start, start base pair of CNV; Stop, stop base pair of CNV; DUP, duplication; DEL, deletion. *Call in unaffected family member, **Call in affected family member from a multiplex family. (DOCX) [file pone.0304514.s011.docx]

| Gene/Region | Chr | Start | Stop | Type |
| --- | --- | --- | --- | --- |
| *KIF1A* | 2 | 241640262 | 241678528 | DUP |
| *KIF1A* | 2 | 241640262 | 241678528 | DUP |
| *KIF1A* | 2 | 241652252 | 241678528 | DUP |
| *KIF1A** | 2 | 241626057 | 241702124 | DUP |
| *KIF1A** | 2 | 241607616 | 241702124 | DUP |
| *KIF1A** | 2 | 241644718 | 241709924 | DUP |
| *LTBP1* | 2 | 32639775 | 33331219 | DUP |
| *LTBP1* | 2 | 32775984 | 33331219 | DUP |
| *LTBP1** | 2 | 32633925 | 33331219 | DUP |
| *LTBP1** | 2 | 32633925 | 33331219 | DUP |
| *LTBP1** | 2 | 32639775 | 33331219 | DUP |
| *RAF1* | 3 | 12599717 | 12803792 | DUP |
| *FLT4** | 5 | 180019198 | 180056863 | DEL |
| *GATA4*** | 8 | 11506208 | 11786255 | DUP |
| *GATA4*** | 8 | 11506208 | 11999394 | DUP |
| *MUC5B* | 11 | 1078312 | 1300406 | DUP |
| *NANOG*** | 12 | 7836807 | 8123777 | DUP |
| *MYH11* | 16 | 14975292 | 16295863 | DUP |
| *MYH11* | 16 | 15484868 | 18309593 | DUP |
| *MAPK3** | 16 | 27977483 | 30174024 | DUP |
| *NCOR1* | 17 | 15976558 | 16012829 | DUP |
| *DSCAM*** | 21 | 41278161 | 41856480 | DUP |
| *DSCAM** | 21 | 41278694 | 41813285 | DUP |
| *TBX1** | 22 | 19682627 | 19755127 | DEL |
| *TBX1* | 22 | 19701341 | 19776365 | DEL |
| *TBX1* | 22 | 19701341 | 19808938 | DEL |
| *CRKL** | 22 | 20742450 | 21461607 | DEL |
