# Supplementary material for: Rare genomic copy number variants implicate new candidate genes for bicuspid aortic valve
Source: PLoS One. 2024 Sep 6;19(9):e0304514. doi: 10.1371/journal.pone.0304514 (PMC11379187; doi:10.1371/journal.pone.0304514)
Supplement: S11 Table — Gene, principal gene/region intersected by CNV; TAA, thoracic aortic aneurysm, TAD, thoracic aortic dissection; AS, aortic stenosis; AR, aortic regurgitation; AVR, aortic valve replacement; Aortic Repair, open or endovascular aortic procedure. (DOCX) [file pone.0304514.s012.docx]

| Gene | Gender | Age | Clinical Presentation | Segregation |
| --- | --- | --- | --- | --- |
| *GJA5* | Male | 26 | - | - |
| *LTBP1* | Female | 44 | Mitral Valve Prolapse | No: CNV in unaffected father and son |
| *LTBP1* | Male | 28 | AR, Aortic Repair | - |
| *KIF1A* | Male | 30 | TAA, AS, AVR, Aortic Repair | - |
| *KIF1A* | Female | 26 | - | Uncertain: CNV not in unaffected father |
| *KIF1A* | Male | - | - | Uncertain: CNV not in unaffected father |
| *RAF1* | Female | 23 | - | - |
| *GATA4* | Female | 31 | AVR | - |
| *GATA4* | Male | 49 | AVR | - |
| *GATA4* | Male | 13 | AVR | - |
| *GATA4* | Female | 24 | TAA | Yes: father and grandfather have CNV and BAV |
| *MYH11* | Male | 67 | AVR | - |
| *MYH11* | Male | 75 | - | - |
| *NCOR1* | Male | 19 | TAA, Aortic Replacement | - |
| 22q11 | Male | 60 | TAA, Other Congenital Lesion, Aortic Repair, AVR | - |
| 22q11 | Male | 65 | - | - |
| 22q11 | Female | 1 | TAA, TAD, AR, AS, AVR, Aortic Repair | - |
| 22q11 | Male | 31 | Mitral Stenosis, Coarctation, Aortic Repair | - |
| *DSCAM* | Female | - | - | Probable: Sibling has CNV and has BAV |
| *DSCAM* | Female | - | - | - |
| *DSCAM* | Male | - | - | - |
| *DSCAM* | Female | - | - | - |
| *CELSR1* | Female | 29 | TAD, Aortic Repair, Other Congenital Lesion | Uncertain: daughter does not have CNV or BAV |
