# Supplementary material for: Rare genomic copy number variants implicate new candidate genes for bicuspid aortic valve
Source: PLoS One. 2024 Sep 6;19(9):e0304514. doi: 10.1371/journal.pone.0304514 (PMC11379187; doi:10.1371/journal.pone.0304514)
Supplement: S12 Table — Chr, chromosome; Start, start base pair of CNV; Stop, stop base pair of CNV; DUP, duplication; DEL, deletion. (DOCX) [file pone.0304514.s013.docx]

| Gene/Region | Chr | Start | Stop | Type |
| --- | --- | --- | --- | --- |
| *GJA5* | 1 | 145723645 | 148343177 | DUP |
| *GJA5* | 1 | 145723739 | 148343177 | DUP |
| *GJA5* | 1 | 145801230 | 147824365 | DUP |
| *GJA5* | 1 | 147166377 | 147308112 | DUP |
| *TMEM87B/FBLN7* | 2 | 110982530 | 113103748 | DUP |
| *TMEM87B/FBLN8* | 2 | 111399346 | 113103748 | DEL |
| *TMEM87B/FBLN9* | 2 | 111404636 | 113215796 | DUP |
| *KIF1A* | 2 | 241623458 | 241697884 | DUP |
| *KIF1A* | 2 | 241623458 | 241697884 | DUP |
| *KIF1A* | 2 | 241623458 | 241698298 | DUP |
| *KIF1A* | 2 | 241623458 | 241724479 | DUP |
| *KIF1A* | 2 | 241626057 | 241689833 | DUP |
| *KIF1A* | 2 | 241626057 | 241689833 | DUP |
| *KIF1A* | 2 | 241626057 | 241689833 | DUP |
| *KIF1A* | 2 | 241626057 | 241689833 | DUP |
| *KIF1A* | 2 | 241626057 | 241689833 | DUP |
| *KIF1A* | 2 | 241626057 | 241702124 | DUP |
| *KIF1A* | 2 | 241626057 | 241702124 | DUP |
| *KIF1A* | 2 | 241640262 | 241689833 | DUP |
| *KIF1A* | 2 | 241640262 | 241697773 | DUP |
| *LTBP1* | 2 | 32619581 | 33299434 | DUP |
| *LTBP1* | 2 | 32619581 | 33331219 | DUP |
| *LTBP1* | 2 | 32633925 | 33302342 | DUP |
| *LTBP1* | 2 | 32633925 | 33302342 | DUP |
| *LTBP1* | 2 | 32633925 | 33331219 | DUP |
| *LTBP1* | 2 | 32633925 | 33331219 | DUP |
| *LTBP1* | 2 | 32633925 | 33331219 | DUP |
| *LTBP1* | 2 | 32633925 | 33369552 | DUP |
| *LTBP1* | 2 | 32689829 | 33331219 | DUP |
| *RAF1* | 3 | 12645681 | 12739194 | DUP |
| *TGFBR2* | 3 | 29993977 | 31273870 | DEL |
| SOX7/GATA4 | 8 | 8064756 | 11882065 | DUP |
| SOX7/GATA4 | 8 | 8064756 | 11882065 | DUP |
| SOX7/GATA4 | 8 | 8064756 | 11882065 | DUP |
| *SOX7/GATA4* | 8 | 8064756 | 12009597 | DUP |
| *SOX7/GATA4* | 8 | 10109379 | 11987960 | DUP |
| *SOX7* | 8 | 10587741 | 10683929 | DEL |
| *GATA4* | 8 | 10914233 | 11853596 | DUP |
| *GATA4* | 8 | 11349186 | 11821835 | DUP |
| *GATA4* | 8 | 11385469 | 11882065 | DUP |
| *TGFBR1* | 9 | 101861767 | 102092282 | DUP |
| *MYH11* | 16 | 14761719 | 16281154 | DUP |
| *MYH11* | 16 | 14761719 | 16315360 | DUP |
| *MYH11* | 16 | 14975292 | 16299148 | DEL |
| *MYH11* | 16 | 14975292 | 16308351 | DUP |
| *MYH11* | 16 | 14975292 | 16308351 | DUP |
| *MYH11* | 16 | 14975292 | 16308351 | DUP |
| *MYH11* | 16 | 14975292 | 16308351 | DUP |
| *MYH11* | 16 | 14975292 | 16308351 | DUP |
| *MYH11* | 16 | 14975292 | 16308351 | DUP |
| *MYH11* | 16 | 14975292 | 16315360 | DUP |
| *MYH11* | 16 | 15092120 | 16291933 | DUP |
| *MYH11* | 16 | 15125441 | 16292128 | DUP |
| *MYH11* | 16 | 15240816 | 18584353 | DUP |
| *MAPK3* | 16 | 29647342 | 30199713 | DUP |
| *MAPK4* | 16 | 29647342 | 30199713 | DUP |
| *MAPK5* | 16 | 29647342 | 30199713 | DUP |
| *DSCAM* | 21 | 41254102 | 41516071 | DUP |
| *DSCAM* | 21 | 41254456 | 41536215 | DUP |
| *TBX1* | 22 | 16874656 | 20241436 | DEL |
| *TBX1* | 22 | 17818807 | 19002159 | DUP |
| *TBX1, CRKL* | 22 | 18644702 | 21726191 | DUP |
| *TBX1, CRKL* | 22 | 18877787 | 21461607 | DUP |
| *TBX1, CRKL* | 22 | 18877787 | 21461607 | DUP |
| *TBX1, CRKL* | 22 | 18877787 | 21028007 | DEL |
| *TBX1, CRKL* | 22 | 18877787 | 21804903 | DEL |
| *TBX1* | 22 | 19062020 | 20264937 | DUP |
| *TBX1* | 22 | 19667336 | 20329526 | DEL |
| *TBX1* | 22 | 19682627 | 20233865 | DEL |
| *TBX1* | 22 | 19682627 | 20262166 | DEL |
| *TBX1* | 22 | 19693418 | 20264937 | DEL |
| *TBX1* | 22 | 19701341 | 20300738 | DEL |
| *TBX1* | 22 | 19724224 | 20300738 | DEL |
| *TBX1, CRKL* | 22 | 19951816 | 24298181 | DUP |
| *CRKL* | 22 | 20719325 | 21726191 | DEL |
| *CRKL* | 22 | 21246902 | 22702508 | DEL |
| *CRKL* | 22 | 21424414 | 22015771 | DUP |
| *CELSR1* | 22 | 45236935 | 48193505 | DEL |
| *CELSR1* | 22 | 46751367 | 47159028 | DUP |
| *CELSR1* | 22 | 46924254 | 46931077 | DEL |
