# Supplementary material for: Rare genomic copy number variants implicate new candidate genes for bicuspid aortic valve
Source: PLoS One. 2024 Sep 6;19(9):e0304514. doi: 10.1371/journal.pone.0304514 (PMC11379187; doi:10.1371/journal.pone.0304514)
Supplement: S13 Table — Chr, chromosome; Start, start base pair of CNV; Stop, stop base pair of CNV; DUP, duplication; DEL, deletion. Some GATA4 CNVs were not identified in the overlap analysis because they exceeded the size threshold (5 Mb). (DOCX) [file pone.0304514.s014.docx]

| Cohort | Gene | Chr | Start | Stop | Type |
| --- | --- | --- | --- | --- | --- |
| EBAV | *GJA5* | 1 | 146326373 | 147340734 | DUP |
| BAVGWAS | *GJA5* | 1 | 145723645 | 148343177 | DUP |
| BAVGWAS | *GJA5* | 1 | 145723739 | 148343177 | DUP |
| BAVGWAS | *GJA5* | 1 | 145801230 | 147824365 | DUP |
| BAVGWAS | *GJA5* | 1 | 147166377 | 147308112 | DUP |
| EBAV | *KIF1A* | 2 | 241640262 | 241678528 | DUP |
| EBAV | *KIF1A* | 2 | 241640262 | 241678528 | DUP |
| EBAV | *KIF1A* | 2 | 241652252 | 241678528 | DUP |
| BAVGWAS | *KIF1A* | 2 | 241623458 | 241697884 | DUP |
| BAVGWAS | *KIF1A* | 2 | 241623458 | 241697884 | DUP |
| BAVGWAS | *KIF1A* | 2 | 241623458 | 241698298 | DUP |
| BAVGWAS | *KIF1A* | 2 | 241623458 | 241724479 | DUP |
| BAVGWAS | *KIF1A* | 2 | 241626057 | 241689833 | DUP |
| BAVGWAS | *KIF1A* | 2 | 241626057 | 241689833 | DUP |
| BAVGWAS | *KIF1A* | 2 | 241626057 | 241689833 | DUP |
| BAVGWAS | *KIF1A* | 2 | 241626057 | 241689833 | DUP |
| BAVGWAS | *KIF1A* | 2 | 241626057 | 241689833 | DUP |
| BAVGWAS | *KIF1A* | 2 | 241626057 | 241702124 | DUP |
| BAVGWAS | *KIF1A* | 2 | 241626057 | 241702124 | DUP |
| BAVGWAS | *KIF1A* | 2 | 241640262 | 241689833 | DUP |
| BAVGWAS | *KIF1A* | 2 | 241640262 | 241697773 | DUP |
| EBAV | *GATA4* | 8 | 11506208 | 11786255 | DUP |
| EBAV | *GATA4* | 8 | 11103895 | 11856864 | DUP |
| EBAV | *GATA4* | 8 | 11448529 | 11808756 | DUP |
| EBAV | *GATA4* | 8 | 11448529 | 11732454 | DUP |
| BAVGWAS | *GATA4* | 8 | 8064756 | 11882065 | DUP |
| BAVGWAS | *GATA4* | 8 | 8064756 | 11882065 | DUP |
| BAVGWAS | *GATA4* | 8 | 8064756 | 11882065 | DUP |
| BAVGWAS | *GATA4* | 8 | 8064756 | 12009597 | DUP |
| BAVGWAS | *GATA4* | 8 | 10109379 | 11987960 | DUP |
| BAVGWAS | *GATA4* | 8 | 10914233 | 11853596 | DUP |
| BAVGWAS | *GATA4* | 8 | 11349186 | 11821835 | DUP |
| BAVGWAS | *GATA4* | 8 | 11385469 | 11882065 | DUP |
| EBAV | *MYH11* | 16 | 14975292 | 16295863 | DUP |
| EBAV | *MYH11* | 16 | 15484868 | 18309593 | DUP |
| BAVGWAS | *MYH11* | 16 | 14761719 | 16281154 | DUP |
| BAVGWAS | *MYH11* | 16 | 14761719 | 16315360 | DUP |
| BAVGWAS | *MYH11* | 16 | 14975292 | 16299148 | DEL |
| BAVGWAS | *MYH11* | 16 | 14975292 | 16308351 | DUP |
| BAVGWAS | *MYH11* | 16 | 14975292 | 16308351 | DUP |
| BAVGWAS | *MYH11* | 16 | 14975292 | 16308351 | DUP |
| BAVGWAS | *MYH11* | 16 | 14975292 | 16308351 | DUP |
| BAVGWAS | *MYH11* | 16 | 14975292 | 16308351 | DUP |
| BAVGWAS | *MYH11* | 16 | 14975292 | 16308351 | DUP |
| BAVGWAS | *MYH11* | 16 | 14975292 | 16315360 | DUP |
| BAVGWAS | *MYH11* | 16 | 15092120 | 16291933 | DUP |
| BAVGWAS | *MYH11* | 16 | 15125441 | 16292128 | DUP |
| BAVGWAS | *MYH11* | 16 | 15240816 | 18584353 | DUP |
| EBAV | *DSCAM* | 21 | 41278694 | 41823356 | DUP |
| EBAV | *DSCAM* | 21 | 41268738 | 41813285 | DUP |
| EBAV | *DSCAM* | 21 | 41278694 | 41823356 | DUP |
| EBAV | *DSCAM* | 21 | 41268738 | 41823356 | DUP |
| BAVGWAS | *DSCAM* | 21 | 41254102 | 41516071 | DUP |
| BAVGWAS | *DSCAM* | 21 | 41254456 | 41536215 | DUP |
| EBAV | *TBX1* | 21 | 18877787 | 21461607 | DUP |
| EBAV | *TBX1* | 21 | 19580050 | 20227551 | DUP |
| EBAV | *TBX1* | 22 | 19701341 | 19776365 | DEL |
| EBAV | *TBX1* | 22 | 19701341 | 19808938 | DEL |
| BAVGWAS | *TBX1* | 22 | 16874656 | 20241436 | DEL |
| BAVGWAS | *TBX1* | 22 | 17818807 | 19002159 | DUP |
| BAVGWAS | *TBX1* | 22 | 18644702 | 21726191 | DUP |
| BAVGWAS | *TBX1* | 22 | 18877787 | 21461607 | DUP |
| BAVGWAS | *TBX1* | 22 | 18877787 | 21461607 | DUP |
| BAVGWAS | *TBX1* | 22 | 18877787 | 21028007 | DEL |
| BAVGWAS | *TBX1* | 22 | 18877787 | 21804903 | DEL |
| EBAV | *CELSR1* | 22 | 46261909 | 51187440 | DEL |
| BAVGWAS | *CELSR1* | 22 | 45236935 | 48193505 | DEL |
| BAVGWAS | *CELSR1* | 22 | 46751367 | 47159028 | DUP |
| BAVGWAS | *CELSR1* | 22 | 46924254 | 46931077 | DEL |
