# Supplementary material for: Rare genomic copy number variants implicate new candidate genes for bicuspid aortic valve
Source: PLoS One. 2024 Sep 6;19(9):e0304514. doi: 10.1371/journal.pone.0304514 (PMC11379187; doi:10.1371/journal.pone.0304514)
Supplement: S14 Table — Chr, chromosome; Start, start base pair of CNV; Stop, stop base pair of CNV; DUP, duplication; DEL, deletion; LOH, loss of heterozygosity. (DOCX) [file pone.0304514.s015.docx]

| Chr | Start | Stop | Type | Description |
| --- | --- | --- | --- | --- |
| 2 | 138066736 | 143331537 | DUP | Mosaic LOH |
| 2 | 183476298 | 189945752 | DUP | Mosaic LOH |
| 3 | 143040791 | 168814375 | DUP | Mosaic LOH |
| 3 | 1 | 7768285 | DEL | Constitutional |
| 6 | 148301116 | 156618923 | DEL | Constitutional |
| 7 | 101355402 | 106892492 | DEL | Mosaic |
| 8 | 6970806 | 12525566 | DUP | Constitutional |
| 8 | 1 | 11987960 | DUP | Constitutional |
| 14 | 101350298 | 107283150 | DUP | Mosaic LOH |
| 14 | 71135027 | 107283150 | DUP | Mosaic LOH |
| 15 | 80465431 | 88497147 | DUP | Mosaic LOH |
| 15 | 93593528 | 102150818 | DUP | Mosaic LOH |
| 15 | 22761722 | 28540261 | DEL | Constitutional |
| 17 | 15175570 | 22234751 | DUP | Mosaic |
| 18 | 67445173 | 78010620 | DEL | Constitutional |
| 20 | 31265482 | 50716159 | DEL | Mosaic |
| 20 | 1 | 25829977 | DEL | Mosaic |
| 20 | 31240778 | 48292606 | DEL | Mosaic |
| 20 | 50320079 | 62960292 | DUP | Constitutional |
| 21 | 14359894 | 48099610 | DUP | Trisomy 21 |
| 21 | 14359894 | 48099610 | DUP | Trisomy 21 |
