# Supplementary figures and images for: Rare genomic copy number variants implicate new candidate genes for bicuspid aortic valve
Source: PLoS One. 2024 Sep 6;19(9):e0304514. doi: 10.1371/journal.pone.0304514 (PMC11379187; doi:10.1371/journal.pone.0304514)

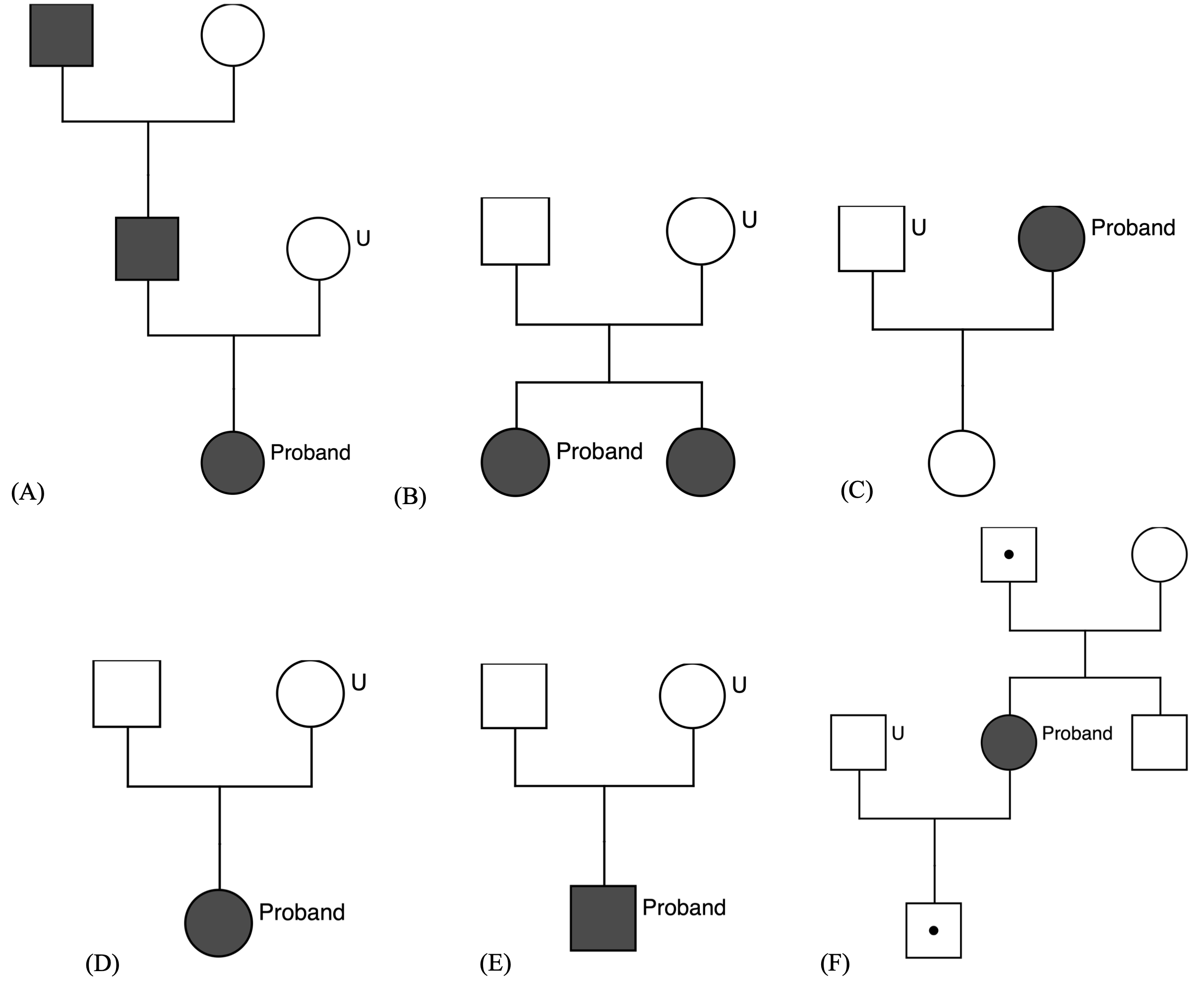

Supplement: S1 Fig — (A) GATA4 CNV; (B) DSCAM CNV; (C) CELSR1 CNV; (D, E) KIF1A CNVs; (F) LTBP1 CNVs. Dot, apparently unaffected CNV carrier; Shaded, affected CNV carrier; U, no genotype was available. (TIFF) [file pone.0304514.s016.tiff]

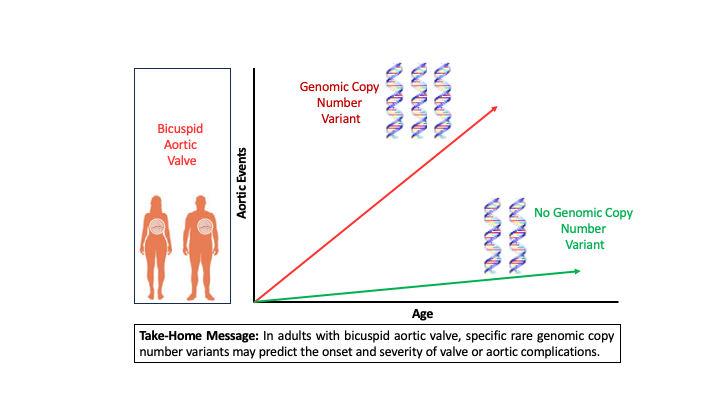

Supplement: S1 File — (PNG) [file pone.0304514.s017.png]
